# A Neonatal Case of Hemorrhagic Shock Due to Congenital Hemangioma

**DOI:** 10.7759/cureus.79719

**Published:** 2025-02-26

**Authors:** Makoto Kudo, Manami Akasaka, Minoru Sakuraba, Yoichi Murakami, Syuji Kusano

**Affiliations:** 1 Pediatrics, Iwate Medical University Hospital, Shiwa, JPN; 2 Paediatrics, Iwate Medical University Hospital, Shiwa, JPN; 3 Plastic and Reconstructive Surgery, Iwate Medical University Hospital, Shiwa, JPN; 4 Pediatrics, Kitakami Saiseikai Hospital, Kitakami, JPN

**Keywords:** congenital hemangioma, hemorrhage, rapidly involuting congenital hemangioma, tachycardia, ulceration, vascular anomalies

## Abstract

Congenital hemangioma (CH) is a rare form of vascular anomaly that develops prenatally, is difficult to differentiate from other vascular anomalies, and poses significant risks, including heart failure and severe hemorrhage. Herein, we present the case of a female infant born with a dark red mass measuring 30 mm × 20 mm in size, located on the right temporal region. She was referred to us for outpatient follow-up but presented to the emergency department on day 21 of life with a massive pulsatile hemorrhage originating from the mass. The patient simultaneously presented with tachycardia and cold extremities. We initiated artificial respiration and compression of the vascular anomalies, and the bleeding was well-controlled. Red blood cell transfusion stabilized her circulation, allowing transfer of the patient to Iwate Medical University Hospital for further evaluation. Owing to difficulties in differentiating CH from other vascular anomalies on imaging, a biopsy was performed. Histological examination revealed a dilated vascular cavity, lined with a single layer of endothelial-like cells with no arterial components. Although hemorrhage from rapidly involutingCH is rare, it is possible that ulceration of the CH could induce hemorrhage.

## Introduction

Vascular tumors and malformations include a variety of vascular anomalies that are often difficult to definitively diagnose in the early stages. These anomalies are defined by the International Society for the Study of Vascular Anomalies classification [1]. Among infants, the incidence of infantile hemangioma (IH) is the highest, occurring in approximately 5-10% of cases [2]. IH typically grows rapidly after birth but subsequently tends to regress spontaneously. By contrast, congenital hemangioma (CH) develops within the uterus, is fully formed at birth, and does not grow [3]. CH is categorized into three types: rapidly involuting CH (RICH), which usually resolves by approximately one year of age; partially involuting CH (PICH), which is partially involuted; and non-involuting CH (NICH), which shows no regression. Differentiating CH from arteriovenous malformations(AVM), kaposiform hemangioendothelioma, and tufted angioma can be challenging. Furthermore, CH can sometimes lead to severe complications such as the Kasabach-Merritt phenomenon and hemorrhage [2], which require careful management.

In general, congenital haemangiomas are very rarely associated with massive hemorrhage.

Herein, we report the case of an infant with CH who developed severe pulsatile hemorrhage from a scalp hemangioma, resulting in hypovolemic shock during follow-up at our outpatient clinic.

## Case presentation

The patient was a female infant who presented on day 21 after birth, with the chief complaint of a mass identified in the right temporal region. Her perinatal history was as follows: gestational age of 38 weeks and six days, birth weight of 2,850 g (0 standard deviation, SD), Apgar score of 9 (one minute) and 10 (five minutes), and negative for all newborn screening tests.

**Figure 1:**
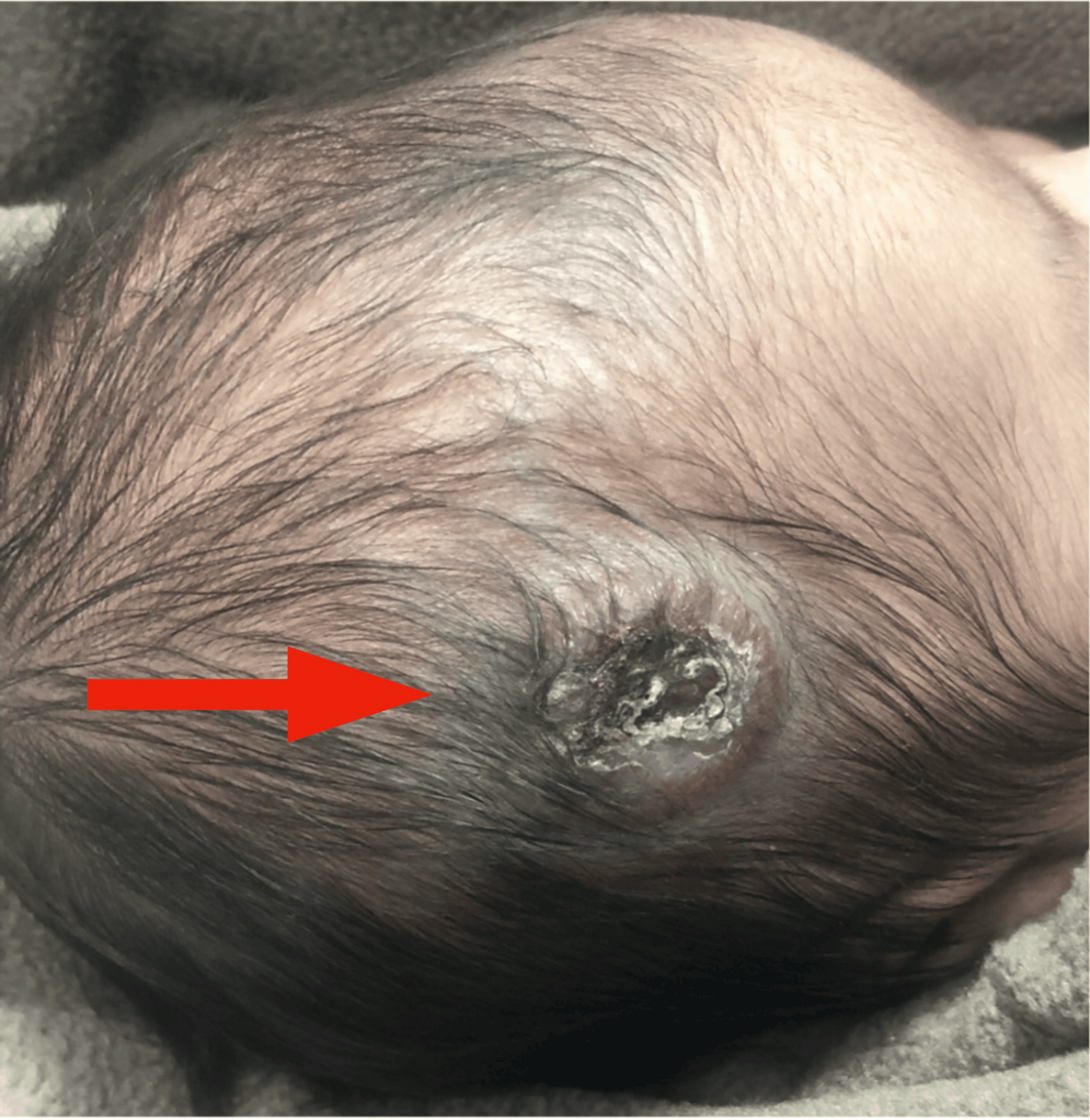
The mass became ulcerated on day 17.

The patient was born via spontaneous vaginal delivery at a local maternity clinic. A mass was noted in the right temporal region at birth, prompting a referral to the Pediatric Department of the referring hospital. Magnetic resonance imaging (MRI) of the head at this time revealed no intracranial or vascular communication, and the patient was initially observed as an outpatient. On day 17, the mass became ulcerated and began to bleed (Figure 1); however, hemostasis was readily achieved, and the patient was admitted for observation. No further bleeding was observed, and the patient was discharged on day 20.

**Figure 2:**
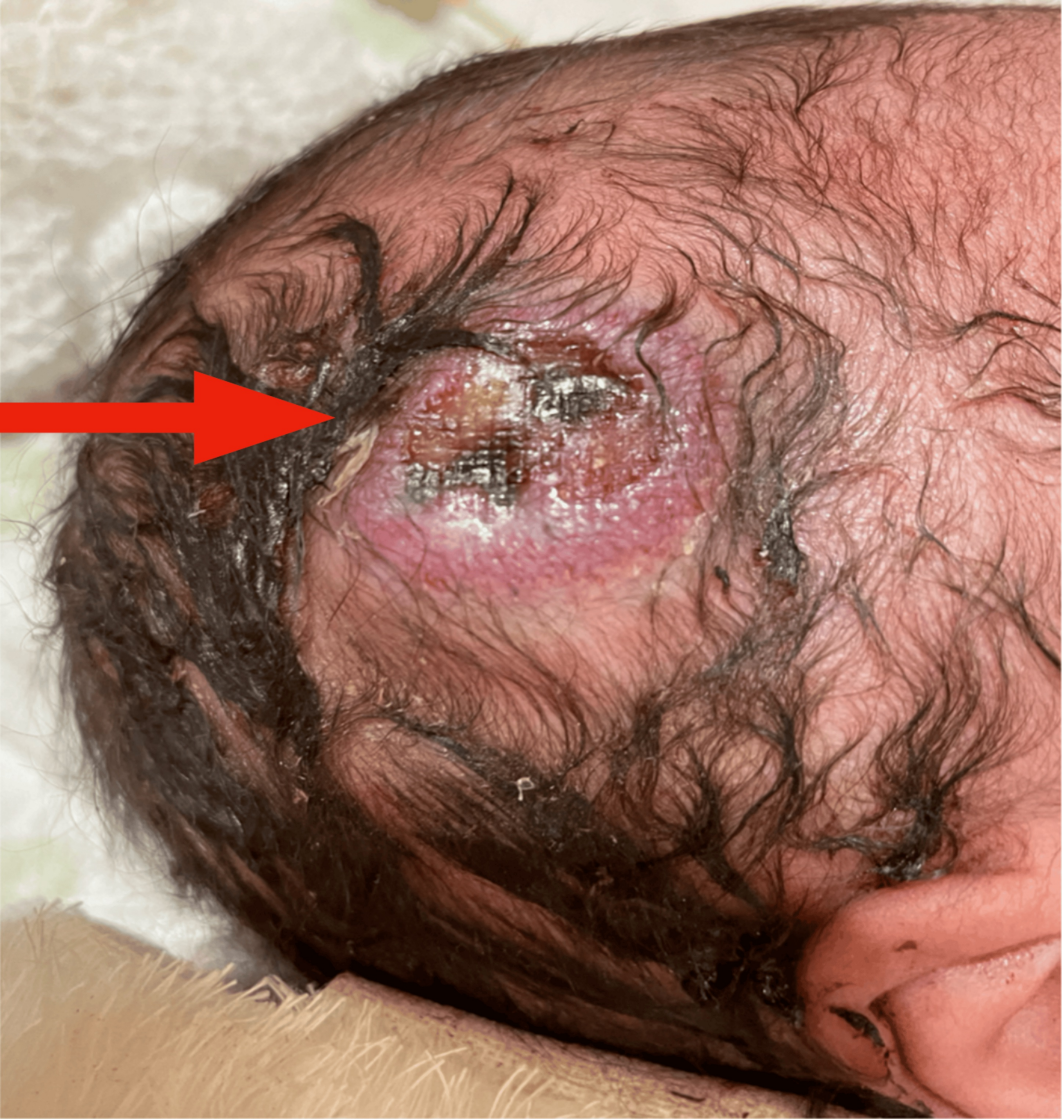
She experienced sudden profuse hemorrhage from the mass. The size was 20 mm× 30 mm in diameter on day 21.

On day 21, she experienced a sudden profuse hemorrhage from the mass, resembling a fountain, and was brought to the emergency department for readmission (Figure 2). The patient was presented to our department with hypovolemic shock and marked anemia (Table 1) and was also referred to the plastic and reconstructive surgery department. Red blood cell transfusion and manual compression of the mass achieved hemostasis after 1 hour. No recurrence of bleeding occurred during hospitalization, and the patient’s general condition improved. Repeat contrast-enhanced computed tomography (CT) and MRI revealed a well-circumscribed mass with vascular components rich in flow voids on T2-weighted MRI. Distinguishing CH from peripheral AVM was difficult. Owing to the pulsatile nature of the hemorrhage, the patient was transferred to Iwate Medical University Hospital on day 30 for a definitive diagnosis.

**Table 1:** Blood test results on day 21.

Blood test	
White blood cell (WBC)	34.1 × 10^3^/μL
Red blood cell (RBC)	1.63 × 10^6^/μL
Hemoglobin (Hb)	5.7 g/dL
Hematocrit (Ht)	16.50%
Platelet (PLT)	515 × 10^3^/μL
Prothrombin time (PT)	10.3 sec
Activated partial thromboplastin time (APTT)	37.4 sec
Fibrinogen (*FBG*)	272.5 mg/dL
AT3	62.6%
Fibrin degradation product (FDP)	<2.5 μg/mL
Lactate dehydrogenase (LDH)	190 U/L
Sodium (Na)	132 mmol/L
Potassium (K)	5.3 mmol/L
Chloride (Cl)	100 mmol/L
Ca	9.8 mg/dL
BUN	10.3 mg/dL
Cre	0.23 mg/dL
IgG	561 mg/dL
IgA	6 mg/dL
IgM	29 mg/dL
CRP	0.55 mg/dL
Fe	39 μg/dL
TIBC	178 μg/dL
UIBC	139 μg/dL
pH	7.287 U/L
pCO2	37.9 mmHg
pO2	21.6 mmHg
HCO3-	17.7 mmol/L
BE	-8.2 mmol/L
BS	188 mg/dL

The patient’s clinical findings on admission were as follows: height, 52.0 cm (-0.3 SD); weight, 3,652 g (-0.9 SD); head circumference, 33 cm; and chest circumference, 36.5 cm. A raised erythematous mass measuring 25 mm, with an ulceration, was present in the right temporal region, with a surrounding 30 mm pulsatile, bluish mass.

**Figure 3:**
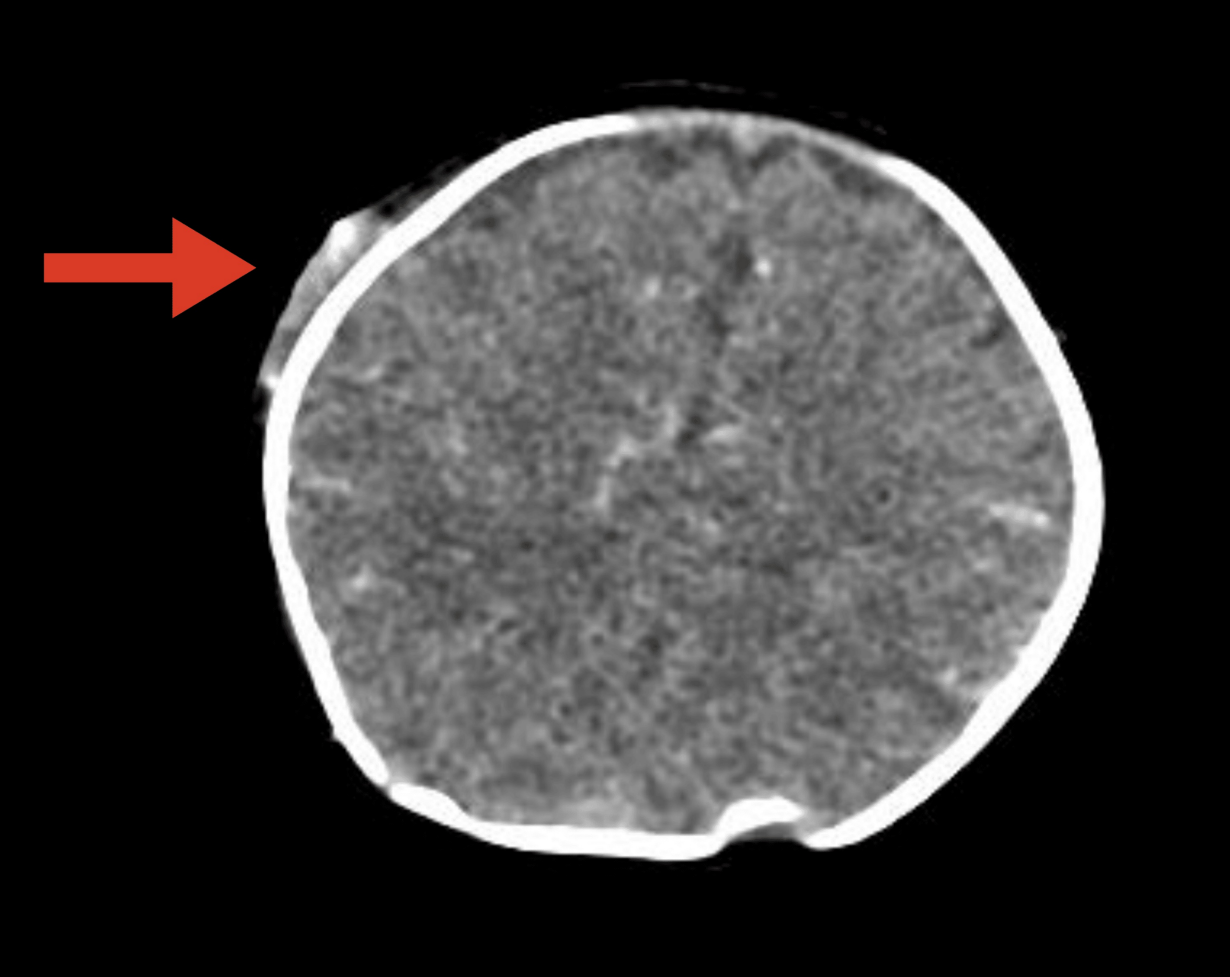
Contrast-enhanced computed tomography showed the mass with rich blood flow component.

**Figure 4:**
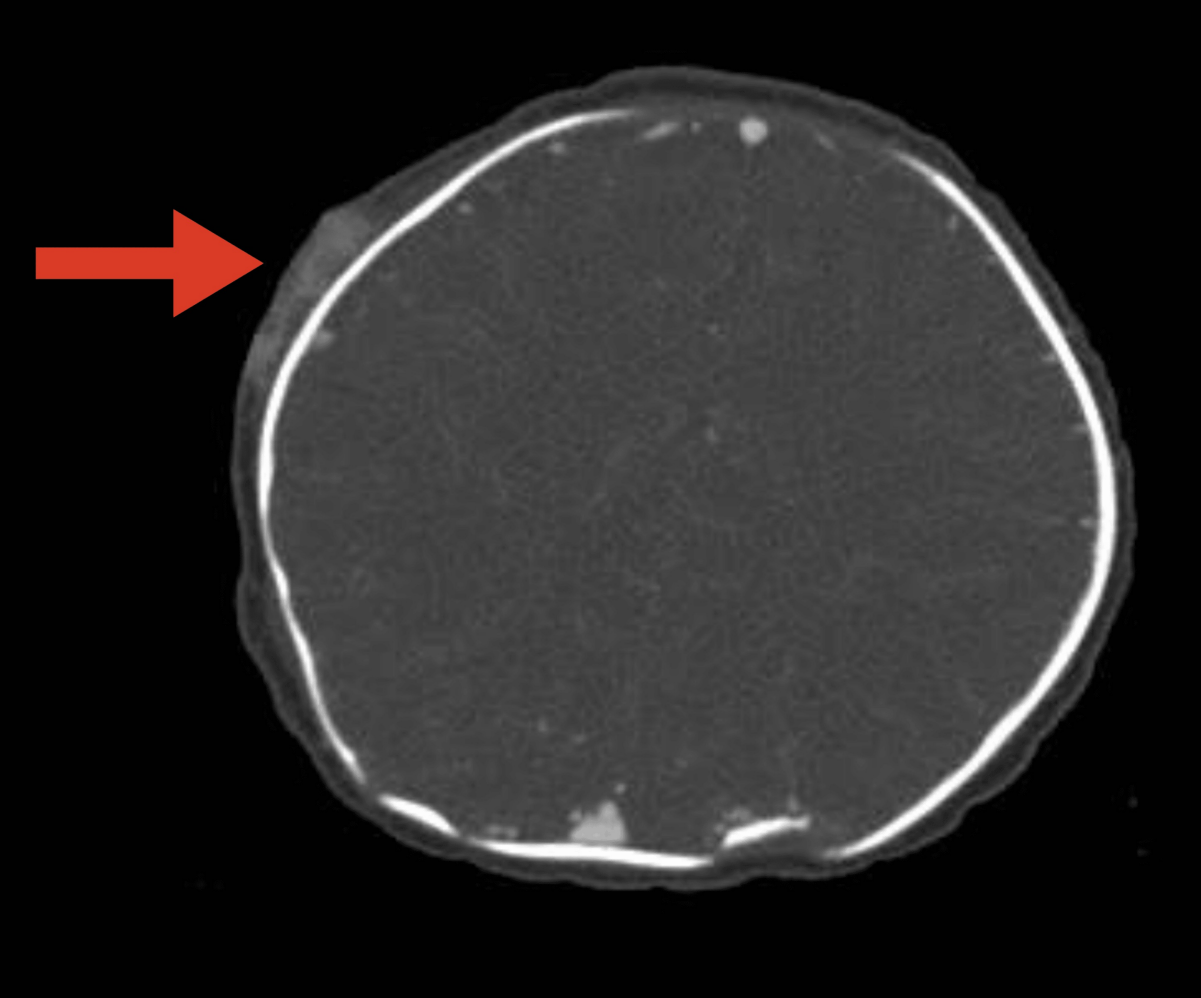
Contrast-enhanced computed tomography showed the mass without bone destruction.

A CT scan of the head (Figures 3-4) showed an enhancing subperiosteal mass with feeding vessels originating from the right superficial temporal artery on CT angiography.

**Figure 5:**
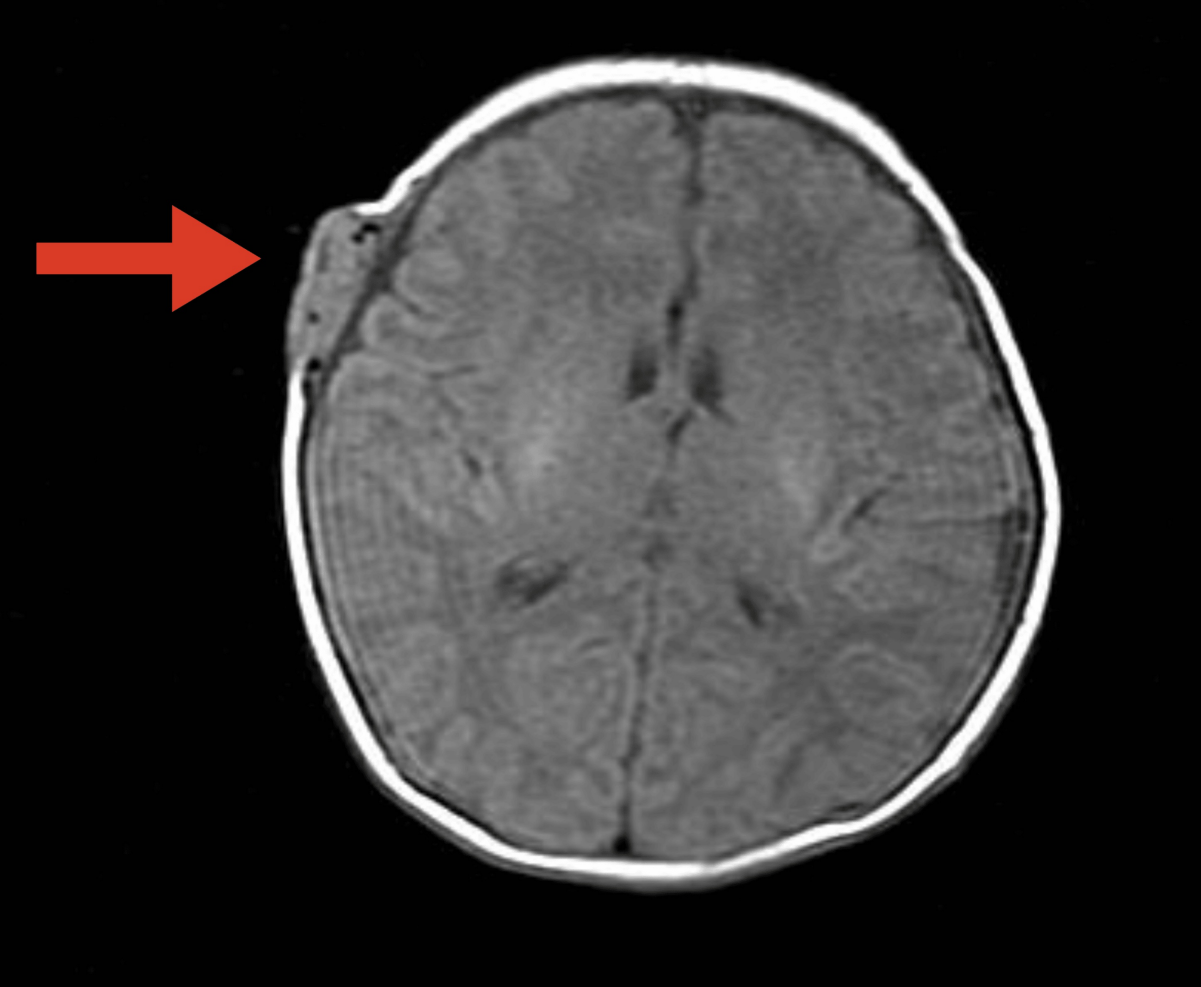
Magnetic resonance imaging with T1-weighted image showed the isosignal mass.

**Figure 6:**
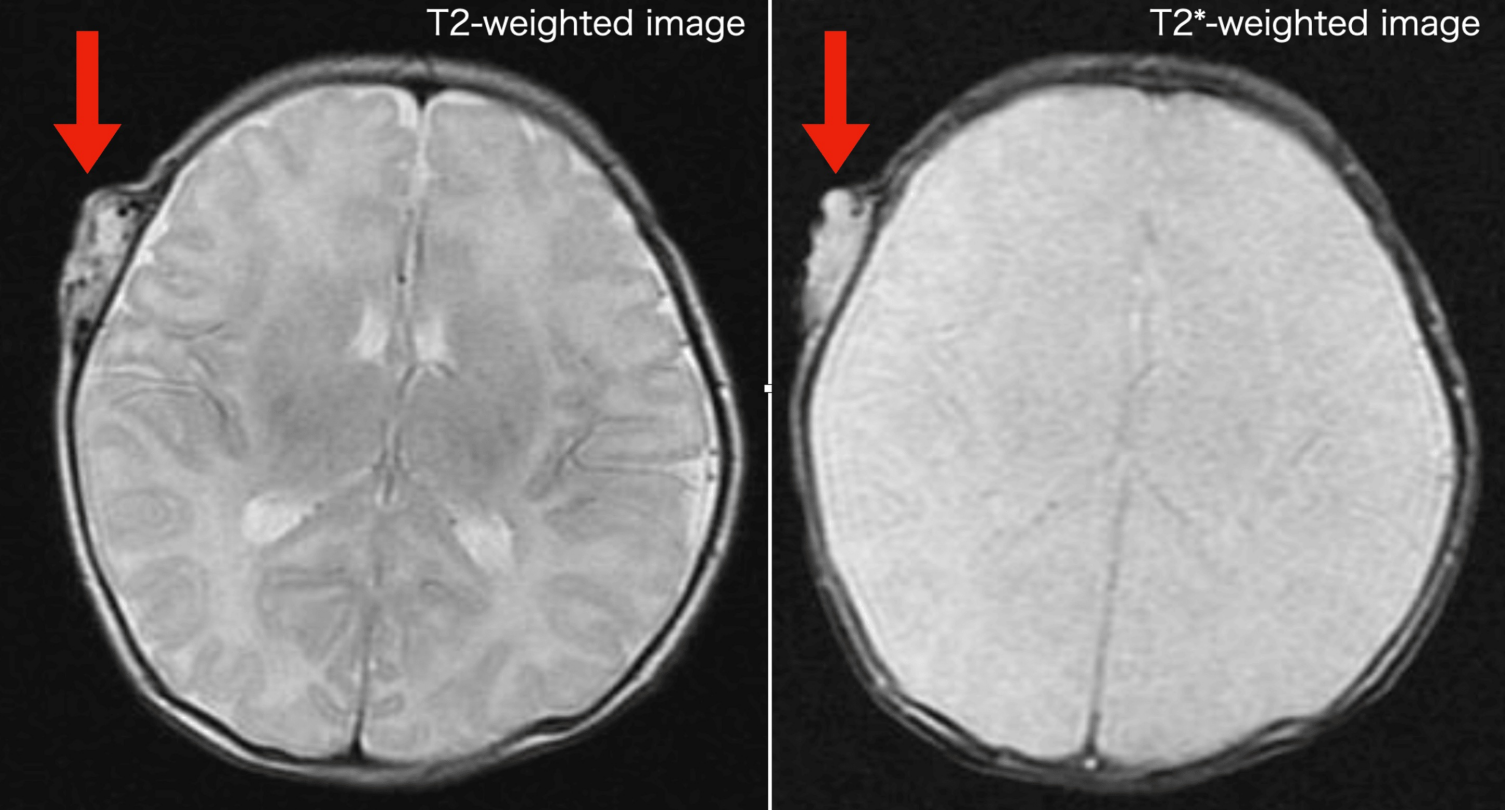
Magnetic resonance imaging with T2-weighted image on the left showed a flow cavity within the mass, with no intracranial or vascular communication. Magnetic resonance imageing with T2*-weighted image on the right showed no flow cavities inside the mass.

An MRI (Figures 5-6) further revealed an isointense signal on T1-weighted images and a hyperintense vascular component with flow voids on T2-weighted images.

**Figure 7:**
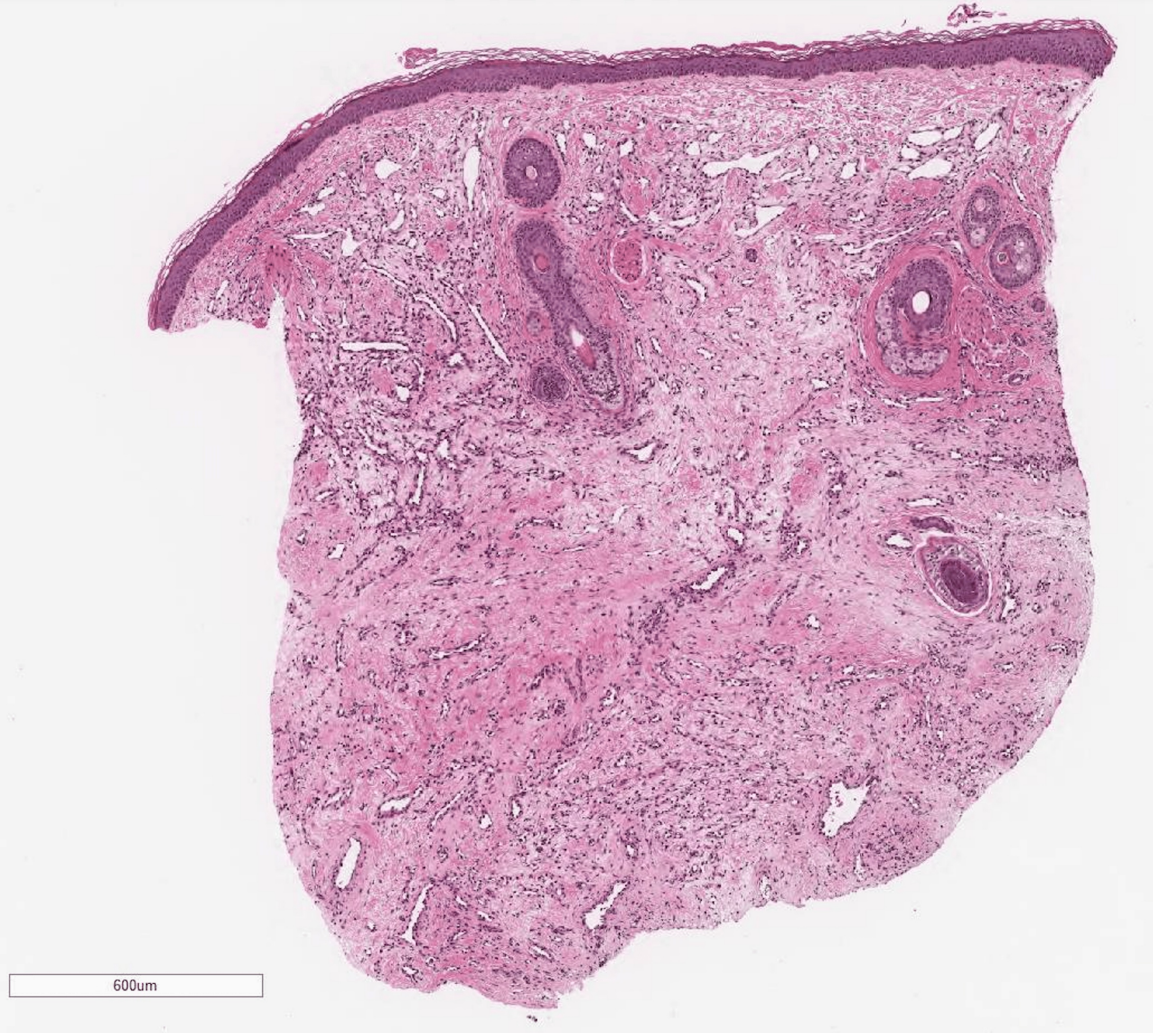
Histological findings revealed a vascular-rich tumor containing intravascular red blood cells with H&E stain.

**Figure 8:**
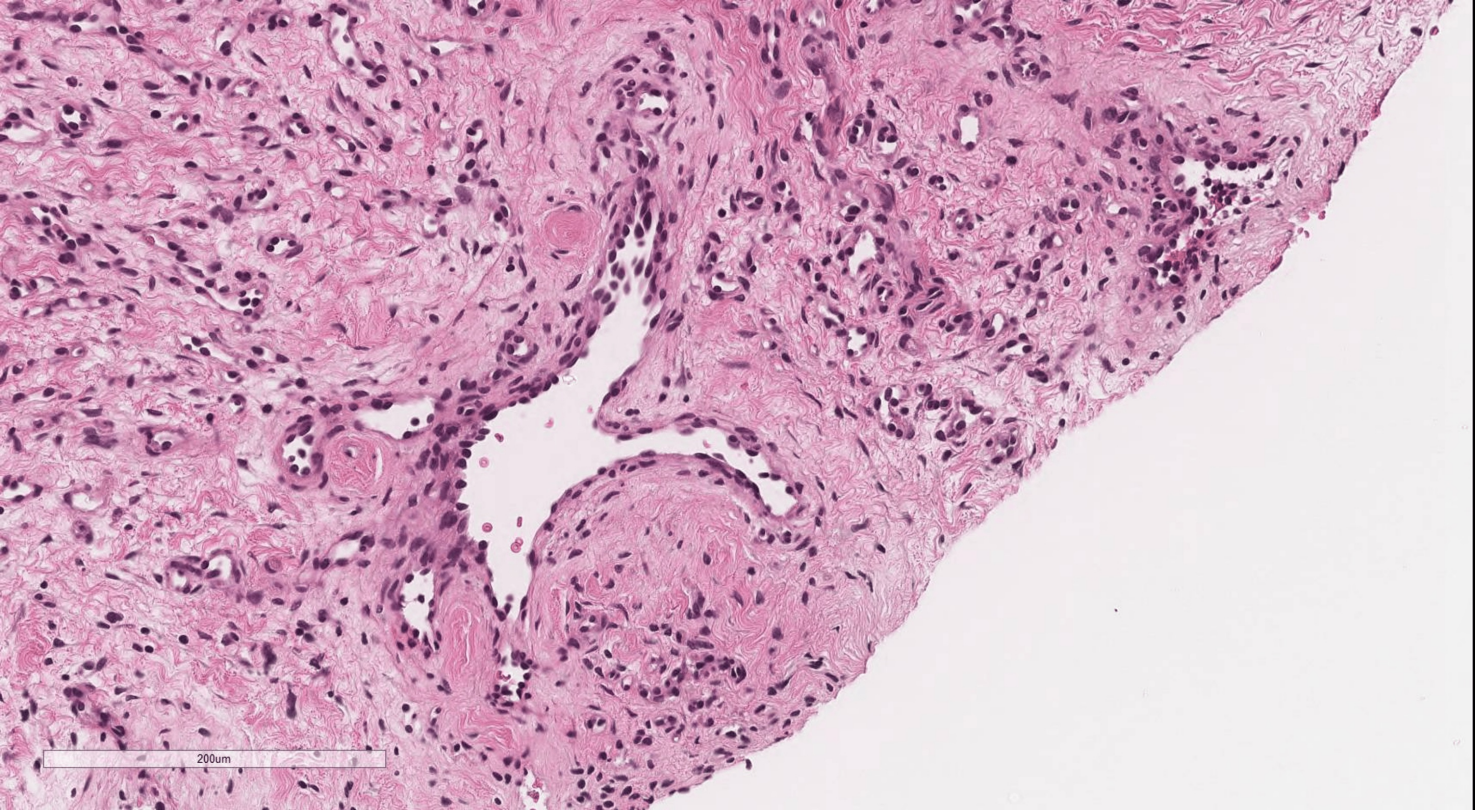
Histological findings revealed a vascular-rich tumor containing intravascular red blood cells with H&E stain.

**Figure 9:**
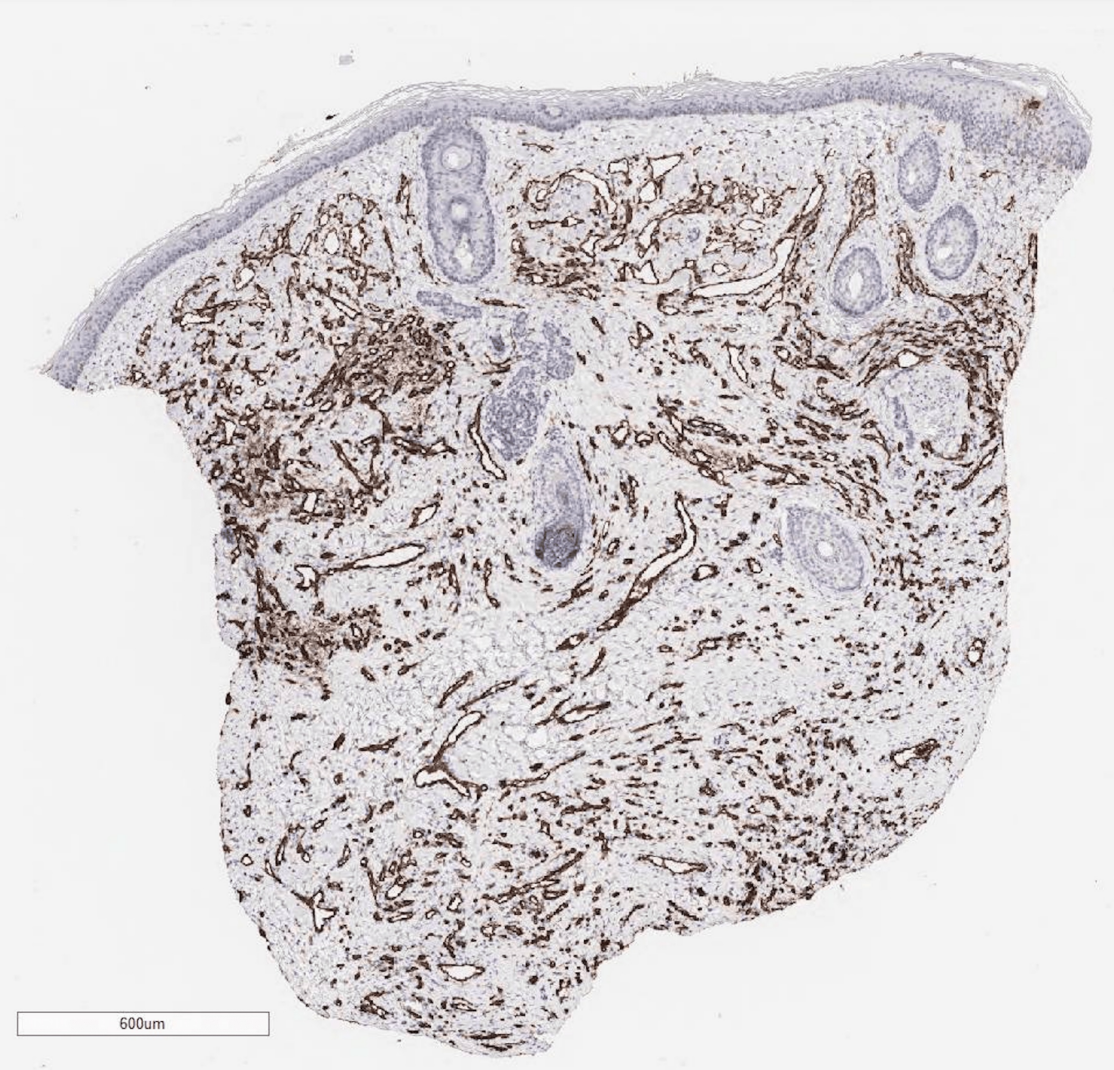
CD31 immunoreactivity is observed in endothelial cells. The vascular spaces are identified by immunohistochemical study for the endothelial marker CD31.

After transfer, the patient experienced no further hemorrhage, maintained oral intake, and demonstrated stable general health. Although AVM was initially suspected on imaging diagnosis, tissue biopsy from the mass was conducted, because of the atypical clinical appearance of the lesion as AVMs. Ultrasonography of the mass prior to biopsy on day 38 revealed a hypoechoic area with interspersed vascular spaces in the center, and inflow from the superficial temporal artery was confirmed. Histological findings revealed a vascular-rich tumor containing intravascular red blood cells with CD31 positivity throughout, indicating a vascular tumor (Figures 7-9).

**Figure 10:**
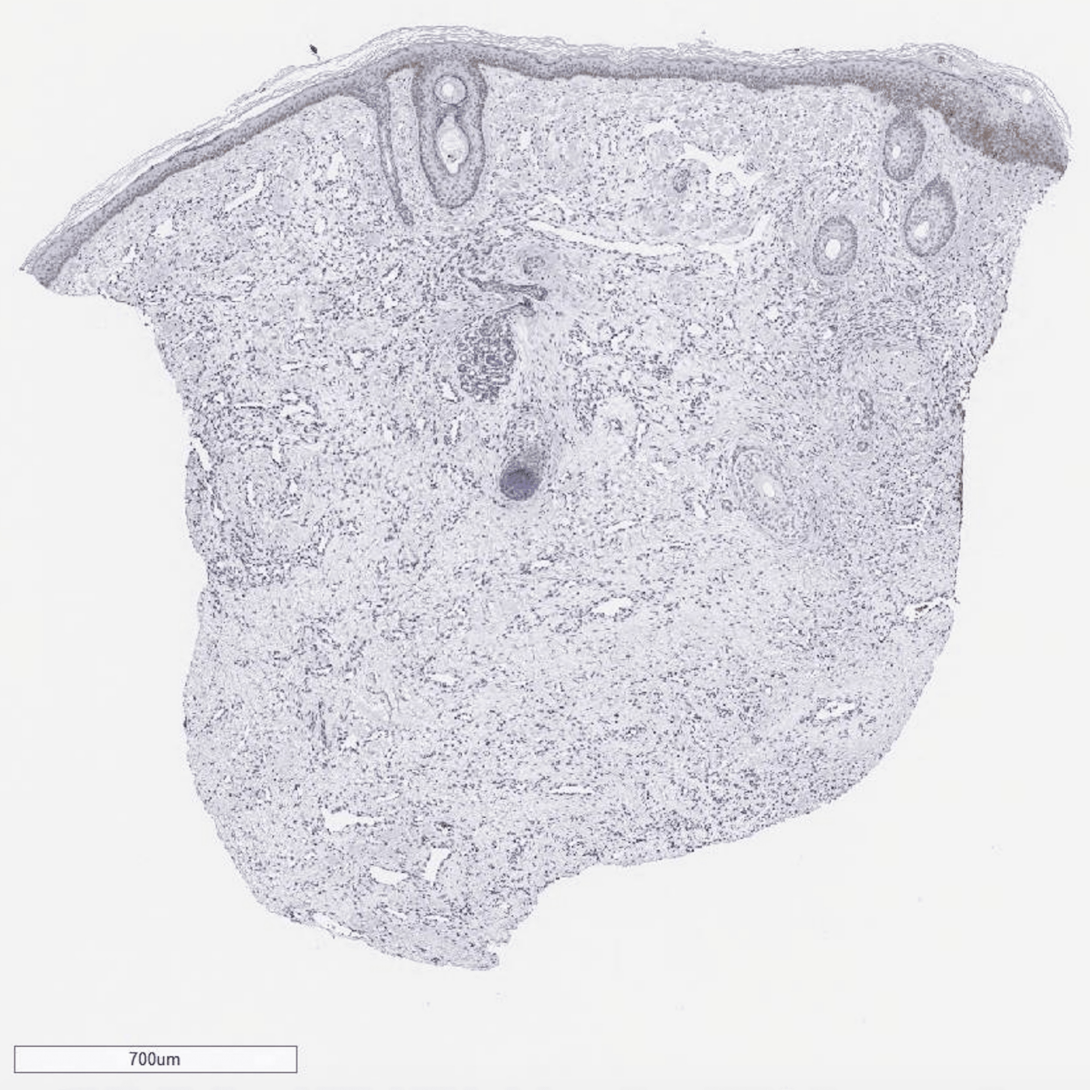
Tissue sections were stained for glucose transporter protein 1(GLUT-1). Tissue vascular endothelial cells were not stained for GLUT-1.

The sections were negative for glucose transporter protein 1 (GLUT1), it is suggested that the vascular tumor is CH (Figure 10).

**Figure 11:**
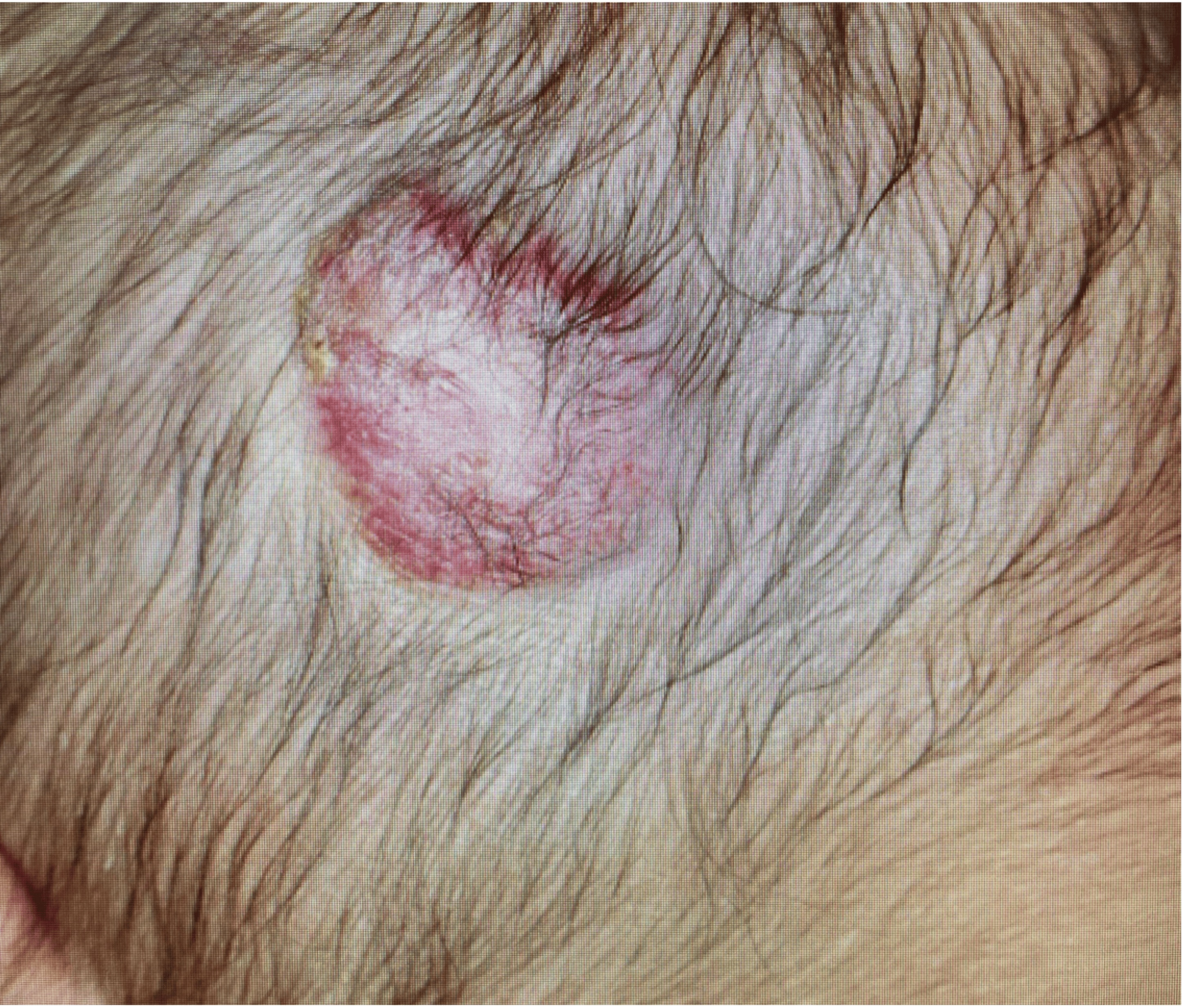
The mass size was decreased to 17 mm× 23 mm in diameter at three months of age.

With no evidence of arterial components or malignancy, the mass was diagnosed as CH. Topical bucillamine sodium ointment was applied to the ulcerated area for three months, leading to gradual resolution. At three months of age, the mass decreased in size to 17 mm × 23 mm (Figure 11). Based on the histological findings and clinical course, a diagnosis of RICH or PICH was made, and the patient remained under observation. Because the parents considered aesthetics with CH to be problematic, at one year of age, she underwent total resection of hemangioma under general anesthesia, and the final pathological diagnosis was also congenital hemangioma, without GLUT1 expression.

## Discussion

Vascular tumors and malformations in neonates present with a wide range of clinical manifestations and diagnoses. Severe complications, such as the Kasabach-Merritt phenomenon and hypovolemic shock due to hemorrhage, can arise in CH. In the present case, hemorrhage from an ulcerated area of the CH resulted in hypovolemic shock.

IH, the most common vascular tumor in infants, typically appears postnatally, transiently increases in size, and subsequently regresses. Histologically, IHs frequently express GLUT-1, LeY, FcγRII, and merosin [4]. These markers, which are also found in the placental microvasculature, indicate a developmental link between IH and placental vasculature. By contrast, CH is a benign congenital vascular tumor with an incidence of approximately 0.2% [5]. At birth, the CH does not grow further but may remain stable or regress. CH can be subdivided into the RICH, PICH, and NICH types based on its rate of involution and generally follows a benign course. However, cases with arteriovenous shunts leading to high-output heart failure [6] or massive hemorrhage [7, 8] have also been reported. Hemorrhage is more likely to occur in patients with ulcerated CH, as has been shown in several cases [6,7].

The present patient exhibited CH with ulceration at birth. Although MRI and contrast-enhanced CT did not yield a conclusive diagnosis, the clinical and histopathological findings indicated RICH or PICH. The mass was nourished by the superficial temporal artery, and the exposed vessels likely contributed to the hemorrhage. Blood tests revealed no abnormalities in the platelet count or coagulation time.

Previous reports have noted hemorrhage from RICH in four cases [6, 7] and from NICH in one case [6].

Tranexamic acid and topical emollients are effective in preventing hemorrhages by stabilizing blood clots [7,9]. Tranexamic acid and ε-aminocaproic acid have so far been used in reports of hemorrhage from congenital hemangioma. Plasmin Inhibitors bind reversibly to plasminogen at the lysine-binding site and inhibit the binding of plasminogen to fibrin and the subsequent degradation of fibrin [10]. In general, Plasmin Inhibitors are expected to inhibit hemorrhage by suppressing plasmin [10]. In previous case reports, plasmin Inhibitors were thought to stabilize the clot locally by limiting endogenous fibrinolysis [7,9].

Residual ulceration can be a risk for hemorrhage in CH. We attempted to prevent hemorrhage by treating ulcers as early as possible. Bucladesin, N6,2‘-O-dibutyryl cyclic 3’,5' adenosine monophosphate sodium, is said to be effective in chronic skin ulcers, including bedsores [11]. Bucladesin crosses cell membranes and is degraded to cAMP by deacetylating enzymes, thereby dilating peripheral blood vessels and improving blood flow obstruction. It also promotes human skin fibroblast proliferation and, together with its angiogenesis-promoting action, promotes granulation. Applied to the ulcer area, the ulcer area disappeared two months after the start of the treatment. For CH hemorrhage, disappearance and compression of the ulcer area are important. The present case suggests that not only transamic acid but also bucladesin is effective.

In the present case, bucladesine sodium ointment resolved the ulcer, potentially preventing rebleeding by promoting granulation and epidermal healing.

## Conclusions

Although CH is a rare tumor with a benign course compared to IH, ulceration is associated with an increased risk of hemorrhage. Therefore, prophylactic measures are essential in ulcerated areas. In certain cases, proactive interventions such as surgical excision or endovascular embolization should be considered. Families should also be educated regarding the potential for hemorrhage, instructed on how to perform pressure hemostasis, and the importance of prompt hospital visits in cases of bleeding.
